# Screening of biomarkers for early diagnosis of trauma-induced coagulopathy based on untargeted metabolomics

**DOI:** 10.3389/fendo.2025.1632694

**Published:** 2025-10-15

**Authors:** Xu Xu, Qianhui Ouyang, Min Shao, Qi Yi, Sixiang Liu, Ying Huang, Jia Wang, Xingwen Zhang, Chaochao Tan

**Affiliations:** ^1^ Department of Clinical Laboratory, Hunan Provincial People’s Hospital (The First Affiliated Hospital of Hunan Normal University), Changsha, Hunan, China; ^2^ School of Medicine, Hunan Normal University, Changsha, Hunan, China; ^3^ Department of Emergency, Hunan Provincial People’s Hospital (The First Affiliated Hospital of Hunan Normal University), Changsha, Hunan, China; ^4^ Department of Research, Hunan Provincial People’s Hospital (The First Affiliated Hospital of Hunan Normal University), Changsha, Hunan, China

**Keywords:** liquid chromatography-tandem mass spectrometry (LC-MS), lysophosphatidylethanolamine (LysoPE), metabolomics, trauma, trauma-induced coagulopathy (TIC)

## Abstract

**Objectives:**

Trauma-induced coagulopathy (TIC) is an acute coagulation disorder characterized by massive bleeding following trauma and is a leading cause of mortality. However, current clinical methods are inadequate for predicting TIC onset, and reliable biomarkers for early diagnosis are lacking. This study aimed to identify potential biomarkers with high sensitivity and specificity for TIC using an untargeted metabolomics approach.

**Methods:**

We analyzed serum samples from 54 trauma patients (27 with TIC and 27 without TIC) and 27 healthy controls. All samples were collected within 24 hours post-trauma. Metabolomic profiling was conducted using liquid chromatography-tandem mass spectrometry (LC-MS).

**Results:**

Metabolite profiles differed significantly between the TIC and non-TIC groups. Two metabolites, LysoPE(20:4(8Z,11Z,14Z,17Z)/0:0) (AUC = 0.933, 95% CI: 0.849–0.995) and LysoPE(0:0/18:2(9Z,12Z)) (AUC = 0.916, 95% CI: 0.818–0.914), were identified as potential biomarkers for distinguishing TIC. The diagnostic performance of these metabolites surpassed that of both conventional coagulation tests and admission assessment scores.

**Conclusion:**

Two LysoPE metabolites were identified as promising biomarkers for the early detection of TIC.

## 1 Introduction

Trauma is a leading cause of mortality worldwide (1). Uncontrolled hemorrhage, which is closely associated with trauma-induced coagulopathy (TIC), is the most common cause of early post-traumatic death (2–4). TIC is a coagulation disorder syndrome occurring in the early phase following trauma (5), characterized initially by a hypocoagulable state presenting as massive bleeding, followed by a late hypercoagulable phase manifesting as excessive coagulation associated with venous thrombosis and multiple organ failure (6). TIC is significantly correlated with increased mortality, greater transfusion requirements, and multiple organ dysfunction (4, 7). Epidemiological studies indicate that approximately 25%-33% of severely injured patients present with TIC upon hospital admission (8). The case fatality rate is four to six times higher than that of patients without coagulation disorders (9), with its pathogenesis involving multiple factors, including endothelial injury, coagulation factor depletion, hyperfibrinolysis, and metabolic disturbances (6).

Clinical diagnosis of TIC primarily relies on conventional coagulation tests (e.g., PT, APTT). While these methods are well-established, they have notable limitations: prolonged turnaround times and an inability to comprehensively assess thrombin generation and fibrinolytic system status (6, 10). Consequently, identifying early diagnostic biomarkers for TIC using metabolomics holds significant promise for improving patient outcomes and reducing mortality (11, 12).

Studies have shown that traumatic brain injury (TBI) is associated with enriched gene expression in coagulation/complement cascades and neuronal markers, as well as elevated levels of glycolytic metabolites and conjugated bile acids (13). In TBI patients with dural invasion, significant increases were observed in blood metabolites involved in late glycolysis, cysteine and one-carbon metabolism, as well as those related to endothelial dysfunction, arginine metabolism, and hypoxia response (14). Currently, no metabolomics studies have focused specifically on whether trauma patients develop TIC. The pathogenesis of TIC leads to alterations in peripheral blood metabolites, and metabolomics research could facilitate further investigation into TIC's pathological mechanisms, potentially uncovering novel mechanisms. Furthermore, no reliable biomarkers are currently available to identify trauma patients who develop TIC, and the associated metabolic pathways remain largely unexplored. Metabolomics, an interdisciplinary approach that systematically analyzes the dynamic changes of small molecule metabolites within biological systems, can reveal aberrant metabolic pathways underlying disease initiation and progression, and has been widely employed to explore diagnostic or prognostic biomarkers for various diseases (15). Existing detection methods fail to predict TIC onset, posing challenges for early diagnosis. The identification of robust biomarkers is crucial for early clinical recognition of TIC patients and improvement of their outcomes. This study employs metabolomics to investigate clinically meaningful biomarkers for early TIC identification. Furthermore, based on differential metabolites, we aim to elucidate significant metabolic pathways involved in TIC pathogenesis.

## 2 Methods

### 2.1 Clinical samples

This study was conducted in accordance with the Declaration of Helsinki and approved by the Ethics Committee of Hunan Provincial People's Hospital, Changsha, China ([2024]-01). Between September 2022 and December 2023, a total of 81 serum samples were collected at the Yuelushan Branch of Hunan Provincial People's Hospital, including 54 from trauma patients (27 with TIC and 27 without TIC) and 27 from healthy controls. All trauma patient samples were obtained within 24 hours after trauma.

This study employed a retrospective case-control design. The case group (TIC group) consisted of all eligible TIC patients (n = 27). To construct a comparable control group, an individual matching strategy was adopted. From trauma patients admitted during the same period who met the inclusion criteria but did not develop TIC, one non-TIC control was matched to each TIC patient based on the following key prognostic factors strongly associated with TIC occurrence: age, diabetes, hypertension, coronary heart disease, and admission scores for trauma patients. However, due to limitations in the available sample pool, matching for sex was not feasible, resulting in a difference in sex distribution between the two groups (**Table 1**).

**Table 1:** Cohort characteristics of trauma-induced coagulopathy (TIC) and non-traumatic coagulopathy (Non-TIC) groups.

Characteristics	TIC (n = 27)	Non-TIC (n = 27)	*P* value
Male, n (%)	15 (55.6%)	22 (81.5%)	0.040*
Age, years, median (P25, P75)	51 (21 - 61)	52 (36 - 59)	0.516
Diabetes, n (%)	2 (7.4%)	3 (11.1%)	1.000
Hypertension, n (%)	8 (29.6%)	3 (11.1%)	0.091
Coronary heart disease, n (%)	2 (7.4%)	0 (0%)	0.471
Hepatitis B, n (%)	1 (3.7%)	4 (14.8%)	0.348
TI trauma index, median (P25, P75)	10 (5 - 12)	8 (5 - 11)	0.424
GCS Coma Index, median (P25, P75)	15 (10 - 15)	15 (14 - 15)	0.070
ISS score, median (P25, P75)	12 (9 - 17)	9 (5 - 18)	0.488
AIS score, median (P25, P75)	3 (3 - 4)	3 (2 - 4)	0.192
PT, median (P25, P75), (S)	14.4 (13.2 - 16.6)	12.6 (12.3 - 14.3)	0.005**
PT-INR, median (P25, P75)	1.19 (1.06 - 1.39)	1.04 (1 - 1.18)	0.005**
PT%, mean ± SD	70.207 ± 25.4751	89.189 ± 14.9870	0.004**
APTT, median (P25, P75), (S)	30.4 (27.2 - 33.2)	26.4 (24.5 - 30.8)	0.04*
TT, median (P25, P75), (S)	12.6 (11.4 - 13.8)	12.6 (11.8 - 13.3)	0.952
FIB, median (P25, P75), (g/L)	1.89 (1.62 - 2.15)	2.01 (1.83 - 2.45)	0.180
DD, mean ± SD, (mg/L)	7102.85 ± 4459.620	6197.56 ± 4283.781	0.378
FDP, median (P25, P75), (mg/L)	31.82 (10.87 - 54.31)	18.41 (9.26 - 35.89)	0.483
AT-3, median (P25, P75)	70.9 (35.8 - 82.8)	88.2 (67.9 - 102.2)	0.045*
K, mean ± SD, (mmol/L)	3.8426 ± 0.78655	3.8925 ± 0.46483	0.431
Na, median (P25, P75), (mmol/L)	139.6 (137 - 143)	139.2 (136.6 - 140.2)	0.188
Cl, median (P25, P75), (mmol/L)	106.8 (102.9 - 109.2)	106.6 (103 - 107.8)	0.979
Ga, median (P25, P75), (mmol/L)	2.17 (1.97 - 2.25)	2.17 (2.09 - 2.24)	0.665
BUN, median (P25, P75), (mmol/L)	4.9 (4.1 - 7.4)	5.8 (4.7 - 6.2)	0.545
Creatinine, median (P25, P75), (umol/L)	70.74 (52.38 - 94.98)	65.98 (54.04 - 79.3)	0.822
Total bilirubin, mean ± SD, (umol/L)	16.233 ± 7.7852	14.311 ± 5.7901	0.308
Direct bilirubin, median (P25, P75), (umol/L)	5.8 (3.4 - 8.0)	4.4 (2.8 - 5.9)	0.117
Indirect bilirubin, mean ± SD, (umol/L)	10.507 ± 5.7432	9.615 ± 3.6842	0.500
ALT, median (P25, P75), (U/L)	21 (17 - 42)	29 (20 - 43)	0.562
AST, median (P25, P75), (U/L)	38 (22 - 122)	32 (23 - 49)	0.441
Death, n (%)	6(22.2%)	2(7.4%)	0.250

*p < 0.05 **p < 0.01.

Diagnostic criteria for TIC (16) (1): Laboratory findings (meeting at least one of the following): PT > 18 s, APTT > 60 s, TT > 15 s, PTr > 1.6; (2) Clinical presentation: Active or potential bleeding requiring blood product transfusion or replacement therapy.

Inclusion criteria: (1) Age 18–80 years; (2) Documented trauma with hemorrhage or blood loss; (3) Time from injury to admission < 24 h; (4) Complete clinical records.

Exclusion criteria: (1) Hematologic disorders or congenital/acquired coagulation abnormalities; (2) Use of corticosteroids, immunosuppressants, or anticoagulants within the past 6 months; (3) Malignancies, severe liver cirrhosis, or other major comorbidities.

The Glasgow Coma Scale (GCS), Trauma Index (TI), Injury Severity Score (ISS) and Abbreviated Injury Scale (AIS) of trauma patients were evaluated upon admission (17–19). Scoring was performed in a single-masked manner by two or more physicians, and the average score was used for analysis.

### 2.2 Sample preparation

Prior to extraction, a mixture of internal standards (10 μL of a cocktail containing stable isotope-labeled compounds such as LPC(17:0)-d5 and PE(17:0/17:0)-d5 at a concentration of 1 μg/mL in methanol) was added to each 100 μL serum aliquot to monitor and correct for variations in sample preparation and instrument analysis. Subsequently, the samples were processed as follows: a 100 μL aliquot, mixed with 400 μL of an 80% methanol aqueous solution, vortexed thoroughly, and incubated on ice for 5 minutes. After centrifugation at 15,000 × g for 20 minutes at 4 °C, the supernatant was collected and diluted with MS-grade water to a final methanol concentration of 53%. A second centrifugation step (15,000 × g, 4 °C, 20 minutes) was performed, and the resulting supernatant was used for LC-MS analysis.

### 2.3 LC-MS analysis

Metabolite profiling was performed using a Vanquish UHPLC system coupled to an Orbitrap Q Exactive™ HF-X mass spectrometer. Samples were injected onto a Hypersil Gold column (100 × 2.1 mm, 1.9 µm) at a flow rate of 0.2 mL/min. In positive ion mode, mobile phase A consisted of 0.1% formic acid in water, while mobile phase B was methanol; in negative ion mode, mobile phase A comprised 5 mM ammonium acetate (pH 9.0) and mobile phase B was methanol. The gradient elution program was as follows: 2% B (0–1.5 min), 2–85% B (1.5–3 min), 85–100% B (3–10 min), 100–2% B (10–10.1 min), and 2% B (10.1–12 min). MS parameters included: spray voltage = 3.5 kV, capillary temperature = 320 °C, sheath gas flow = 35 psi, auxiliary gas flow = 10 L/min, S-lens RF level = 60, and auxiliary gas heater temperature = 350 °C.

### 2.4 Data processing and quality assurance

The raw data files were processed using Compound Discoverer (CD) 3.1 software. Metabolic peaks were aligned across samples with tolerances of ≤ 0.2 minutes for retention time (RT) deviation and ≤ 5 ppm for mass deviation. Peak extraction criteria included a mass deviation of 5 ppm, signal intensity deviation of 30%, and a signal-to-noise ratio (S/N) ≥ 3. Ion peaks with more than 50% missing values within sample groups were removed from the dataset. The remaining missing values were imputed with half of the minimum detected value.

Molecular formulas were predicted based on molecular ions and fragment ions, and matched against the mzCloud (https://www.mzcloud.org/), mzVault, and a custom local database. A matching score higher than 36 out of 60 was required for the MS/MS fragment ion spectra to enhance identification confidence.

To ensure data quality and instrumental stability, a pooled quality control (QC) sample was prepared by combining equal aliquots (10 μL) from every individual serum sample in the study. This QC sample was injected repeatedly at the beginning of the analytical sequence to condition the system, and then after every 10 experimental samples throughout the run. The relative standard deviations (RSDs) of the peak areas for the detected metabolites in the QC samples were calculated. Metabolites with an RSD > 30% in the QC samples were considered unstable and were removed from subsequent data analysis, ensuring the reliability of the dataset. The repeated analysis of the same QC sample throughout the run served as a technical replicate to assess the reproducibility of the entire analytical platform.

Subsequent statistical data processing was performed on a Linux (CentOS 6.6) platform using R and Python. Finally, data from positive and negative ion modes were merged into a comprehensive data matrix containing all relevant features extracted from the raw spectra. All subsequent statistical analyses were conducted using this integrated dataset.

### 2.5 Statistical analysis

Clinical data were analyzed using SPSS 26. Normally distributed continuous variables were expressed as mean ± SD and compared using independent t-tests. Non-normally distributed data were presented as median (P25, P75) and analyzed using nonparametric tests. Categorical variables were reported as counts (%) and compared via chi-square tests. A two-tailed P < 0.05 was considered statistically significant.

To further evaluate the independence of the identified differential metabolites, multivariable logistic regression analysis was performed. The analysis used TIC status as the dependent variable and was adjusted for the confounding factor of sex. After adjusting for this potential confounder, both LysoPE(20:4(8Z,11Z,14Z,17Z)/0:0) and LysoPE(0:0/18:2(9Z,12Z)) remained significant independent predictors of TIC (P < 0.05). (see **Appendix, Tables 1, 2**).

### 2.6 Metabolomics analysis

The data matrix was uploaded to MetaboAnalyst 6.0 (https://www.metaboanalyst.ca/). Data preprocessing on the MetaboAnalyst platform included: normalization by the sum method to adjust for systematic differences; log10 transformation to approximate a normal distribution; and Pareto scaling. Subsequently, t-tests, fold-change (FC) analysis, and volcano plot generation were conducted, along with the construction of PCA, PLS-DA, and OPLS-DA models. The data matrix was then imported into SIMCA14.1 software for a 200-permutation test of the OPLS-DA model. Finally, ROC curve analysis and metabolic pathway analysis were performed on the MetaboAnalyst 6.0 platform. Leave-one-out cross-validation (LOOCV) was performed using R software (version 4.4.3) to obtain an unbiased estimate of the AUC and avoid overoptimism.

## 3 Results

### 3.1 Study cohort and patient characteristics

The clinical characteristics of the trauma group (Tra) and control group (H) are presented in **Table 2**, with no significant differences observed between the groups. The clinical features of the trauma-induced coagulopathy (TIC) and non-TIC cohorts are shown in **Table 1**. Notably, the TIC group contained significantly fewer male patients. No significant differences were observed in the incidence of other underlying conditions or in the admission assessment scores between the groups.

**Table 2:** Cohort characteristics of trauma (Tra) and healthy control (H) groups.

Characteristics	Tra (n = 54)	H (n = 27)	*P* value
Male, n (%)	37 (68.5%)	14 (51.9%)	0.143
Age, years, median (P25, P75)	51 (32 - 59.25)	47 (31 - 55)	0.630
Diabetes, n (%)	5 (9.3%)	1 (3.7%)	0.653
Hypertension, n (%)	11 (20.4%)	7 (25.9%)	0.571
Coronary heart disease, n (%)	2 (3.7%)	0 (0.0%)	0.550
Hepatitis B, n (%)	5 (9.3%)	4 (14.8%)	0.708

### 3.2 Metabolomics study

A total of 81 serum samples were analyzed, comprising 27 from TIC patients, 27 from non-TIC trauma patients, and 27 from healthy participants. Liquid chromatography–mass spectrometry (LC-MS) detected 7,433 metabolic features and identified 1,479 metabolites.

**Figure 1:**
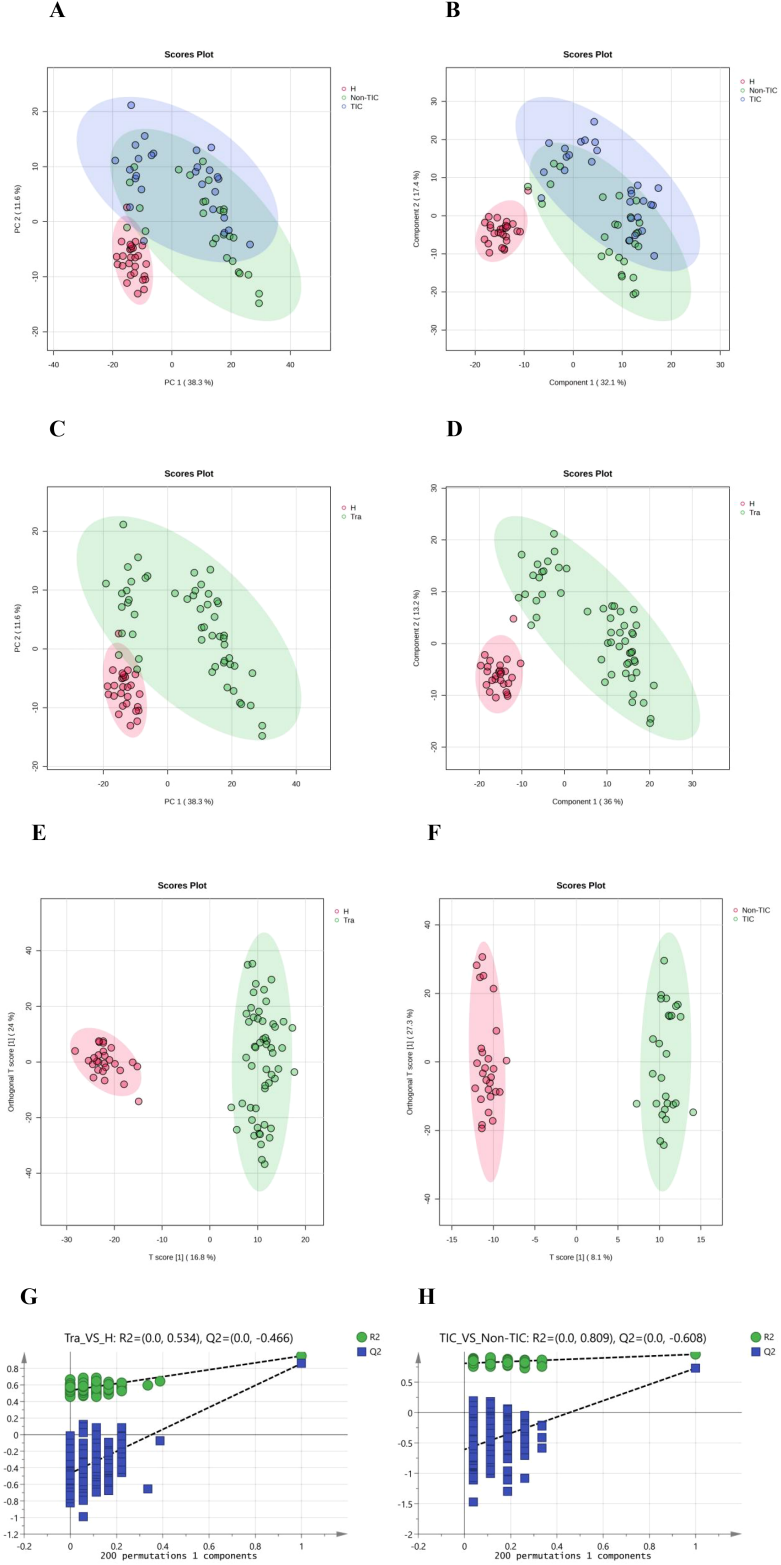
Multivariate statistical analysis of the three groups: **(A)** Principal Component Analysis (PCA), **(B)** Partial Least Squares Discriminant Analysis (PLS-DA); Multivariate statistical analysis of the Tra and H groups: **(C)** Principal Component Analysis (PCA), **(D)** Partial Least Squares Discriminant Analysis (PLS-DA), **(E)** Orthogonal Partial Least Squares Discriminant Analysis (OPLS-DA), **(G)** 200 permutation tests of OPLS-DA; Multivariate statistical analysis of the TIC and Non-TIC groups: **(F)** Orthogonal Partial Least Squares Discriminant Analysis (OPLS-DA), **(H)** 200 permutation tests of OPLS-DA.

Preprocessed data were imported into MetaboAnalyst 6.0 for multivariate pattern analysis. Principal component analysis (PCA) revealed small intra-group but significant inter-group differences in serum metabolite profiles among all three groups (**Figure 1A**). Partial least squares-discriminant analysis (PLS-DA), a supervised method, enhanced inter-group separation while minimizing intra-group variation, demonstrating distinct metabolomic patterns across groups (**Figure 1B**).

PCA, PLS-DA, and orthogonal PLS-DA (OPLS-DA) models consistently showed minimal intra-group but marked inter-group differences between the trauma (Tra) and healthy control (H) groups (**Figures 1C–E**). OPLS-DA further improved model specificity by filtering out classification-unrelated noise. Application of OPLS-DA to the TIC versus non-TIC comparison revealed clear separation between the groups, indicating significant metabolomic disparities (**Figure 1F**). A 200-permutation test confirmed the reliability of all OPLS-DA models (**Figures 1G, H**). Metabolites with variable importance in projection (VIP) scores >1.5 were selected as biologically significant.

### 3.3 Analysis of differential metabolites

**Figure 2:**
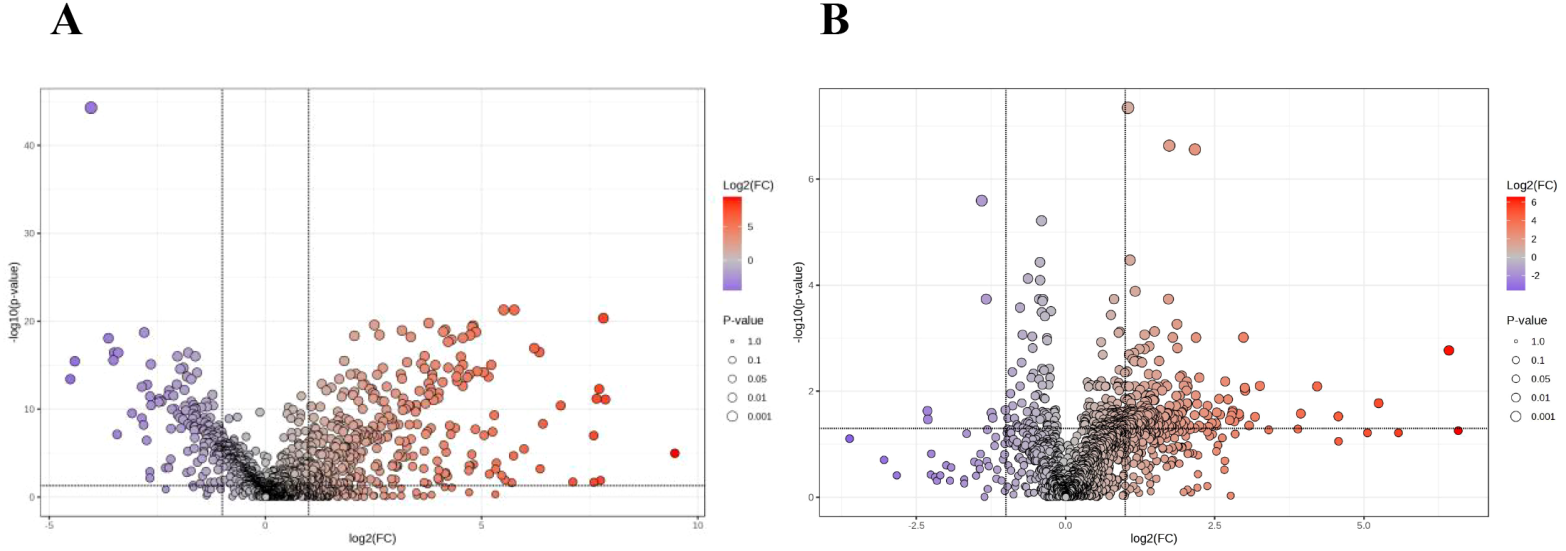
The volcano plot of the Tra and H groups **(A)**, and the volcano plot of the TIC and Non-TIC groups **(B)**; The X-axis corresponds to log 2 (FC), and the Y-axis corresponds to -log 10 (P value).

Student's t-test and fold-change (FC) analysis were performed on the metabolic data of the Tra and H groups, as well as the TIC and non-TIC groups, and volcano plots were generated. Differential metabolites between the two groups were identified. Based on FC > 2 and adjusted P < 0.05, 496 differential metabolites were screened in the Tra and H groups, of which 144 were significantly down-regulated and 352 were significantly up-regulated (**Figure 2A**). In the TIC and non-TIC groups, 212 differential metabolites were identified, of which 7 were significantly down-regulated and 205 were significantly up-regulated (**Figure 2B**).

By integrating multivariate and univariate analyses (FC > 2.0, adjusted P < 0.05, and VIP > 1.5), we identified 186 differential metabolites between the Tra and H groups (69 down-regulated, 117 up-regulated). For the TIC versus non-TIC comparison, 74 metabolites were selected (2 down-regulated, 72 up-regulated).

### 3.4 Metabolic pathway analysis

**Figure 3:**
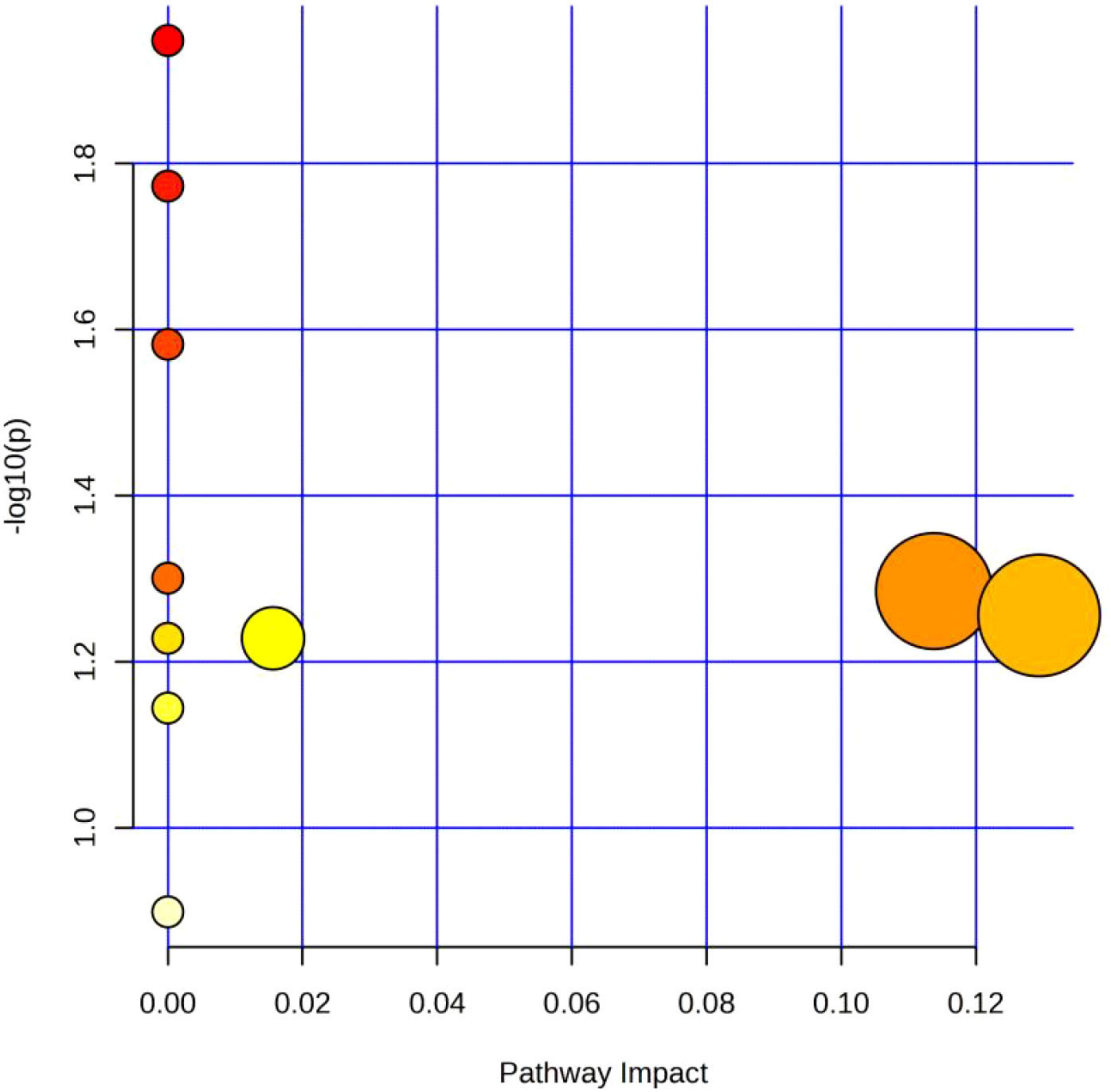
Metabolic pathway analysis diagram of the TIC and Non-TIC groups; The X-axis corresponds to Pathway Impact, and the Y-axis corresponds to -log 10 (P). The large circle on the left represents Alanine, aspartate and glutamate metabolism, and the one on the right represents Inositol phosphate metabolism.

Next, receiver operating characteristic (ROC) curve analysis was used to evaluate the diagnostic efficacy of the differential metabolites. The area under the curve (AUC) values of the 74 differential metabolites for distinguishing the TIC and non-TIC groups were all ≥ 0.7 (see appendix). To elucidate the potential association between these differential metabolites and traumatic coagulopathy, metabolic pathway analysis was performed on the screened differential metabolites using MetaboAnalyst 6.0. Using -log10(p) > 1.0 and Pathway Impact > 0.10 as screening criteria, the analysis revealed that the key metabolic pathways differentiating the TIC and non-TIC groups were inositol phosphate metabolism and alanine, aspartate, and glutamate metabolism (**Figure 3**).

### 3.5 Screening of potential biomarkers

**Figure 4:**
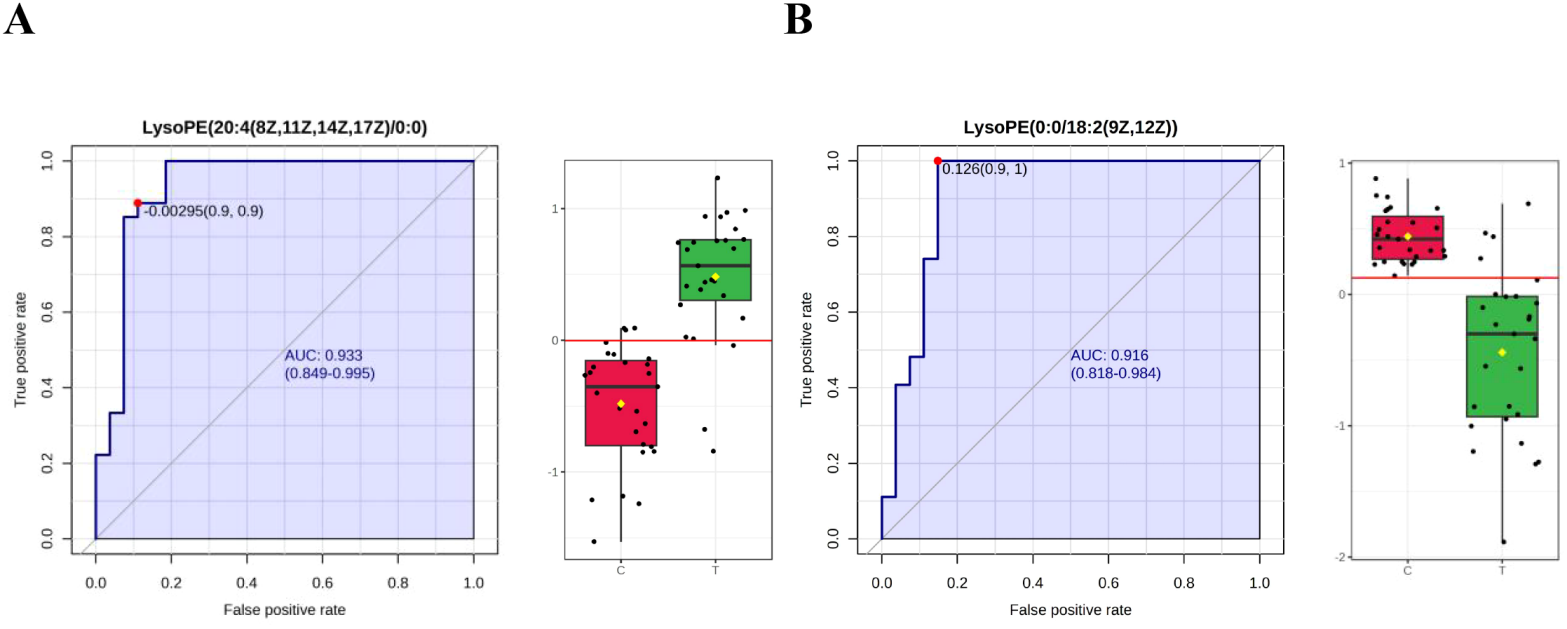
ROC curves of potential biomarkers in the TIC and Non-TIC groups **(A)** LysoPE(20:4(8Z,11Z,14Z,17Z)/0:0), **(B)** LysoPE(0:0/18:2(9Z,12Z)); Through the ROC curve analysis, the diagnostic value of different metabolites in disease screening was evaluated. The x-axis and y-axis represent the false positive rate and the true positive rate respectively.

Using FC > 2.0, adjusted P < 0.05, VIP > 1.5, and AUC > 0.9 as criteria, five differential metabolites were identified as candidate biomarkers for distinguishing TIC from non-TIC. After excluding three exogenous compounds, two metabolites—LysoPE(20:4(8Z,11Z,14Z,17Z)/0:0) and LysoPE(0:0/18:2(9Z,12Z))—were selected as potential biomarkers with high diagnostic value (**Figures 4A, B**).

LysoPE(20:4(8Z,11Z,14Z,17Z)/0:0) yielded an AUC of 0.933 (95% CI: 0.849–0.995), with a sensitivity and specificity of 0.963 and 0.815, respectively. LysoPE(0:0/18:2(9Z,12Z)) had an AUC of 0.916 (95% CI: 0.818–0.914), with a sensitivity and specificity of 0.963 and 0.852, respectively (**Table 3**). Leave-one-out cross-validation yielded the following AUC values: 0.929 for LysoPE(20:4(8Z,11Z,14Z,17Z)/0:0) and 0.885 for LysoPE(0:0/18:2(9Z,12Z)) (see **Appendix**; **Figure 1**).

**Table 3:** Performance characteristics of biomarkers and routine coagulation indicators.

Indicator	Cut-off	Sensitivity	Specificity	AUC (95% cl)	*P*	Youden index
LysoPE(20:4(8Z,11Z,14Z,17Z)/0:0)	0.092	0.963	0.815	0.933 (0.849, 0.995)	< 0.01	0.778
LysoPE(0:0/18:2(9Z,12Z))	0.185	0.963	0.852	0.916 (0.818, 0.914)	< 0.01	0.815
PT	12.78	0.852	0.519	0.724 (0.587, 0.860)	0.005	0.371
PT-INR	1.045	0.852	0.519	0.725 (0.588, 0.862)	0.005	0.371
PT%	63.85	0.519	0.074	0.272 (0.135, 0.409)	0.004	0.407
APTT	27.15	0.778	0.556	0.663 (0.517, 0.808)	0.040	0.334

Current admission assessments for trauma patients—including the Trauma Index (TI), Glasgow Coma Scale (GCS), Injury Severity Score (ISS), and Abbreviated Injury Scale (AIS)—showed no statistical differences between TIC and non-TIC groups (P > 0.05) and demonstrated poor ability to predict TIC onset. Presently, the clinical diagnosis of TIC primarily relies on conventional coagulation tests such as PT and APTT.

**Figure 5:**
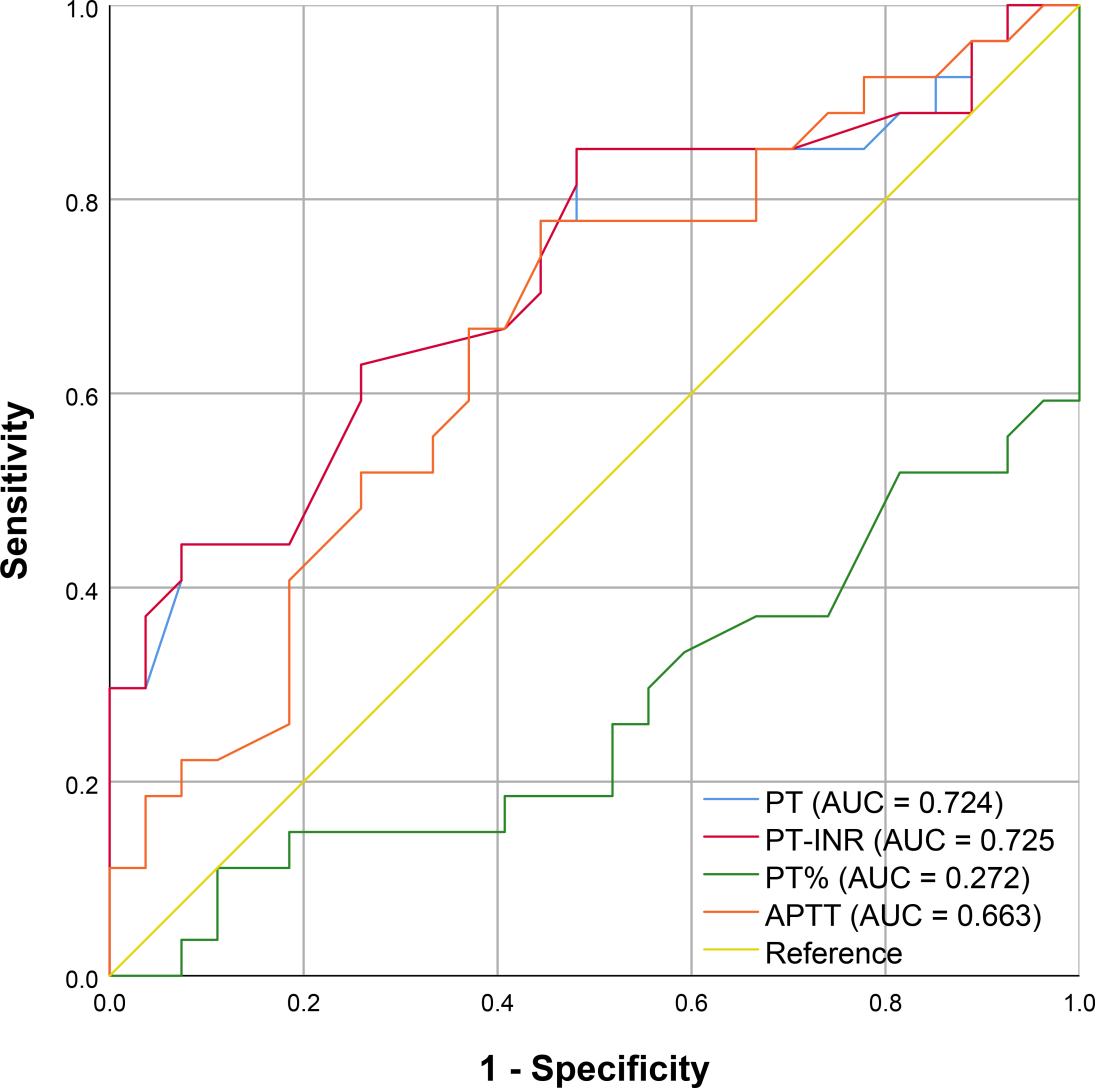
ROC curve of routine coagulation indexes; The X-axis corresponds to 1 - Specificity, and the Y-axis corresponds to Sensitivity.

Although PT, PT-INR, PT%, and APTT values differed significantly between TIC and non-TIC groups (P < 0.05), their diagnostic efficacy was markedly inferior to that of the two differential metabolites identified in this study (**Figure 5**). These metabolites demonstrated significantly higher sensitivity and specificity, as well as superior overall diagnostic performance (**Table 3**). The AUC values of LysoPE(20:4(8Z,11Z,14Z,17Z)/0:0) and LysoPE(0:0/18:2(9Z,12Z)) were significantly higher than those of PT, PT-INR, PT%, and APTT (P < 0.05).

## 4 Discussion

Although conventional coagulation tests such as prothrombin time (PT) and activated partial thromboplastin time (APTT) remain the standard diagnostic tools for trauma-induced coagulopathy (TIC) in clinical practice, these parameters lack the sensitivity to detect early TIC onset and fail to reliably predict subsequent coagulation deterioration. Consequently, there is an urgent unmet need for clinical methods to anticipate TIC development in trauma patients. This study aimed to address this need by employing a metabolomics approach to identify highly sensitive and specific biomarkers for the early diagnosis of TIC.

Our metabolomic analysis revealed that two lysophosphatidylethanolamines—LysoPE(20:4(8Z,11Z,14Z,17Z)/0:0) and LysoPE(0:0/18:2(9Z,12Z))—serve as novel biomarkers for TIC, demonstrating exceptional diagnostic performance in early-stage detection. These metabolites represent novel quantitative indicators for TIC diagnosis, exhibiting significantly higher AUC values, sensitivity, and specificity than traditional coagulation assays (**Table 3**). The superior diagnostic accuracy of these biomarkers (AUC > 0.9) highlights their potential to revolutionize clinical practice by enabling earlier intervention, thereby improving patient outcomes. Furthermore, their high predictive capacity suggests utility in identifying at-risk patients prior to overt coagulopathy, facilitating preemptive therapeutic strategies. Additionally, inositol phosphate metabolism and alanine, aspartate, and glutamate metabolism were also associated with TIC.

LysoPE(20:4(8Z,11Z,14Z,17Z)/0:0) and LysoPE(0:0/18:2(9Z,12Z)) are two distinct lysophosphatidylethanolamine (LysoPE) molecules. Previous research on increased cardiovascular disease risk under hypercholesterolemia found that the oxidized low-density lipoprotein (ox-LDL) group exhibited significantly reduced APTT and PT, along with markedly elevated levels of seven LysoPEs in plasma metabolomics (20). Our observation of significantly elevated serum levels of LysoPE(20:4(8Z,11Z,14Z,17Z)/0:0) in patients with TIC provides direct evidence that an increase in LysoPE can lead to coagulation dysfunction, supporting its potential role in inducing a hypercoagulable state. However, LysoPE(0:0/18:2(9Z,12Z)) levels were significantly decreased in TIC patient plasma, a phenomenon that may be closely related to the essential pathogenesis of TIC. Specifically, TIC progresses acutely through different pathological stages (hypocoagulability, hyperfibrinolysis, and hypercoagulability), with transitions from hypocoagulability to hypercoagulability potentially occurring within minutes or hours. These dynamic changes result in a unique mixed bleeding-thrombosis phenotype throughout the disease course.

Metabolic pathway analysis revealed that the most significantly altered pathways in the TIC/non-TIC comparison were inositol phosphate metabolism and alanine, aspartate, and glutamate metabolism. The inositol phosphate metabolism pathway generates inositol trisphosphate (IP_3_), which promotes the release of intracellular stored calcium ions and extracellular calcium influx, thereby increasing intracellular calcium concentration (21, 22). Elevated calcium levels lead to platelet activation and thrombosis. Existing studies have demonstrated the important role of inositol phosphate metabolism in TIC pathogenesis (22, 23). Our results further support the upregulation of this pathway in TIC patients, suggesting its contribution to coagulation abnormalities.

This was a single-center study with a relatively limited sample size, which may restrict the generalizability and statistical power of our findings. For example, the gender difference between the TIC and non-TIC groups likely resulted from the limited sample size. Although the case-control design employed is suitable for early-stage biomarker screening, it does not allow for an accurate evaluation of the biomarker's actual diagnostic performance (such as sensitivity and specificity) in a consecutively enrolled prospective cohort. This study preliminarily identified two LysoPEs that exhibited significant alterations in TIC patients and demonstrated promising discriminative ability in the initial cohort. External validation in larger independent cohorts is warranted to further assess their diagnostic efficacy and clinical applicability. Furthermore, all samples were collected within 24 hours post-trauma, which precluded continuous dynamic monitoring of the two LysoPEs and thus limited the exploration of their time-dependent dynamic characteristics. The specific pathways and functional roles by which the differential metabolites influence coagulation function will need to be further validated through *in vitro* experiments, such as endothelial cell models. Moreover, the cost of detecting the two LysoPEs currently offers no distinct advantage over routine coagulation tests, and their use has not been widely adopted in clinical practice.

By exploring metabolomic differences in emergency severe trauma patients with or without TIC, we identified potential biomarkers with excellent diagnostic value and determined key metabolic pathways that may play crucial roles in disease progression. This study lays the foundation for future research, which should involve multiple clinical centers and larger sample sizes, as well as integrate transcriptomics and proteomics to elucidate the metabolic-coagulation interaction network and conduct more in-depth investigations.

## Data Availability

The raw data supporting the conclusions of this article will be made available by the authors, without undue reservation.
