# Supplementary material for: Screening of biomarkers for early diagnosis of trauma-induced coagulopathy based on untargeted metabolomics
Source: Front Endocrinol (Lausanne). 2025 Oct 15;16:1632694. doi: 10.3389/fendo.2025.1632694 (PMC12568411; doi:10.3389/fendo.2025.1632694)
Supplement: Supplementary file 2 [file Table1.docx]

Table 1 Multivariate modeling

|  | B | SE | Wald | P | Exp(B) | 95% CI |
| --- | --- | --- | --- | --- | --- | --- |
| Male | -2.027 | 1.108 | 3.347 | 0.067 | 0.132 | 0.015~1.156 |
| LysoPE(20:4(8Z,11Z,14Z,17Z)/0:0) | -3.318 | 0.934 | 12.613 | <0.001 | 0.036 | 0.006~0.226 |
| constant | 2.669 | 1.442 | 3.425 | 0.064 | 14.422 |  |

Table 2 Multivariate modeling

|  | B | SE | Wald | P | Exp(B) | 95% CI |
| --- | --- | --- | --- | --- | --- | --- |
| Male | -1.224 | 0.961 | 1.624 | 0.203 | 0.294 | 0.045~1.933 |
| LysoPE(0:0/18:2(9Z,12Z)) | 3.66 | 1.159 | 9.966 | 0.002 | 38.862 | 4.006~377.046 |
| constant | 0.655 | 1.316 | 0.248 | 0.619 | 1.925 |  |

**A B**


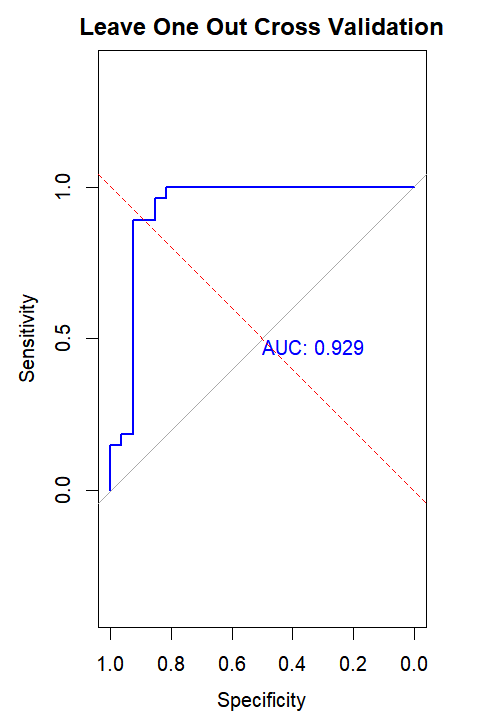

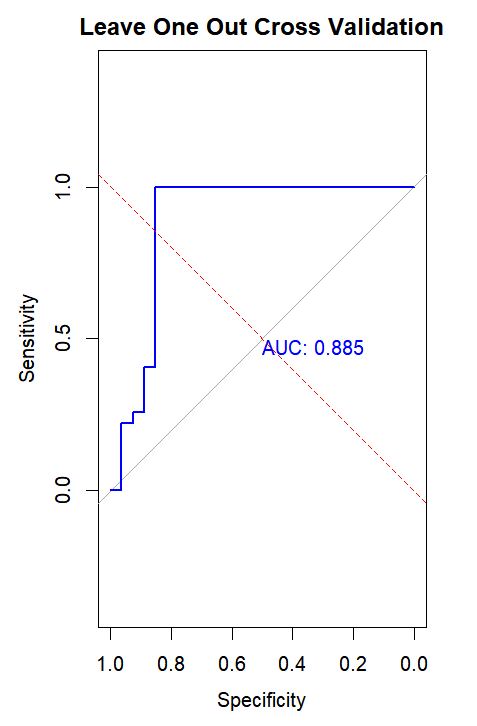


Figure 1 AUC value of leave-one-out cross-validation (A) LysoPE(20:4(8Z,11Z,14Z,17Z)/0:0), (B) LysoPE(0:0/18:2(9Z,12Z)); The X-axis corresponds to Specificity, and the Y-axis corresponds to Sensitivity.
